# Detection Rates of Mild Cognitive Impairment in Primary Care for the United States Medicare Population

**DOI:** 10.14283/jpad.2023.131

**Published:** 2023-10-24

**Authors:** Ying Liu, H. Jun, A. Becker, C. Wallick, S. Mattke

**Affiliations:** 1 Center for Economic and Social Research, University of Southern California, 635 Downey Way, Los Angeles, CA 90089 USA; 2 Department of Health Care Policy, Harvard Medical School, Boston, MA USA; 3 Center for Improving Chronic Illness Care, University of Southern California, Los Angeles, CA USA; 4 US Medical Affairs, Genentech, Inc., South San Francisco, CA USA

**Keywords:** Alzheimer’s disease, detection, mild cognitive impairment, dementia

## Abstract

**Background:**

Existing evidence points to substantial gaps in detecting mild cognitive impairment in primary care but is based on limited or self-reported data. The recent emergence of disease-modifying treatments for the Alzheimer’s disease, the most common etiology of mild cognitive impairment, calls for a systematic assessment of detection rates in primary care.

**Objectives:**

The current study aims to examine detection rates for mild cognitive impairment among primary care clinicians and practices in the United States using Medicare claims and encounter data.

**Design:**

Observational study.

**Setting:**

Medicare administrative data.

**Participants:**

The study sample includes a total of 226,756 primary care clinicians and 54,597 practices that had at least 25 patients aged 65 or older, who were enrolled in Medicare fee-for-service or a Medicare Advantage plan between 2017 and 2019.

**Measurements:**

The detection rate for mild cognitive impairment is assessed as the ratio between the observed diagnosis rate of a clinician or practice as documented in the data, and the expected rate based on a predictive model.

**Results:**

The average detection rates for mild cognitive impairment is 0.08 (interquartile range=0.00–0.02) for both clinicians and practices, suggesting that only about 8% of expected cases were diagnosed on average. Only 0.1% of clinicians and practices had diagnosis rates within the expected range.

**Conclusions:**

Mild cognitive impairment is vastly underdiagnosed, pointing to an urgent need to improve early detection in primary care.

**Electronic Supplementary Material:**

Supplementary material is available for this article at 10.14283/jpad.2023.131 and is accessible for authorized users.

## Introduction

**A**lzheimer’s disease (AD) is a progressive neurodegenerative disorder and the most common etiology of dementia (1). After numerous failed clinical trials, the recent publication (2) of the phase 3 trial results of lecanemab in early-stage AD, followed by FDA’s full approval (3) on July 6, 2023, and the release of positive trial results for donanemab (4) provided the conclusive proof that removing from the brain beta-amyloid deposits, which are hypothesized to be on the critical path of the disease’s pathology, reduces the progression of the disease. This emergence of disease-modifying treatment options represents a paradigm shift for the field, as only symptomatic treatments were available thus far. Those treatments, however, are being released into a poorly prepared healthcare system (5), as the long absence of targeted therapies implies the lack of a robust ecosystem for diagnosis and delivery.

These amyloid-directed treatments are ideally used in early disease-stages, at the stage of mild cognitive impairment (MCI) and no later than mild dementia (6), because they slow down disease progression but cannot reverse decline. This preventive paradigm creates the necessity to detect cases with mild or no subjective symptoms, typically in primary care settings, and to refer them to AD specialists for a formal diagnosis and determination of treatment eligibility. While several studies have pointed out that insufficient numbers of AD specialists and imaging facilities may delay patients’ access to treatment (5), little is known about how well primary care physicians are prepared to identify patients with early-stage AD to initiate those referrals.

The existing evidence for detection of MCI in primary care points to substantial gaps in diagnosis based on self-report. White et al (7) analyzed data from the Health and Retirement Study (HRS), a longitudinal panel survey of older U.S. adults that contains cognitive assessments data and self-reported diagnoses, and found that only 11% of individuals with incident MCI reported having received a diagnosis of cognitive impairment. Similarly, Savva et al (8) determined on the basis of neuropsychiatric testing data in the Aging, Demographics, and Memory Study that only 15% of participants with a Clinical Dementia Rating of 0.5, a score reflective of MCI, were aware of a diagnosis of cognitive impairment. These estimates are consistent with the findings of a German study (9) that primary care physicians correctly identified only 11–12% of cases with established mild cognitive impairment. Additionally, among those who were referred to AD specialists for evaluation, Thoits et al (10) found that about 79% of 110 randomly selected patients had already progressed to moderate or severe dementia, which would be outside of the window (6) for disease-modifying AD treatments.

These aforementioned studies are often limited by their study sample or data source, especially in contrast to the much more robust literature on dementia detection, which has compared diagnoses in claims data with those on death certificates (11), cognitive testing surveys (12), and clinical diagnoses in a dementia registry (13), and largely found acceptable agreement in detection rates. To our knowledge, no study has analyzed documented diagnoses of MCI in claims and encounter data.

Against this background, the current study aims to estimate the contemporary detection rates of MCI in the full Medicare population for U.S. primary care clinicians and practices. We compare their expected numbers of MCI cases, based on a predictive model, to the actually diagnosed cases as documented in claims and encounter data to estimate detection rates between 2017 and 2019. We also account for the uncertainty in these estimates to determine whether detection rates are within the expected range or significantly higher or lower.

## Methods

### Patient and Clinician Samples from Medicare Data

The data used in this study include the enrollment data, as well as claims and encounter data for inpatient and outpatient facilities, carriers, and skilled nursing facilities between 2017 and 2019 for the 100% Medicare sample. Our analysis was restricted to beneficiaries aged 65 and older who were nearly continuously enrolled in Medicare fee-for-service or a Medicare Advantage Plan. Following the coverage definition used by the Chronic Conditions Data Warehouse (CCW), our definition of *nearly continuous enrollment* requires an average of 11 months of both Part A and B or Part C coverage each year (at least 33 out of a possible 36 months); if the beneficiary died during the third year of the surveillance period, it requires fully continuous Part A and B or Part C coverage and no interruption until the month of death. This restriction excluded about 13% of beneficiaries.

Next, we restricted the beneficiaries to those who had office visits, identified based on Evaluation & Management codes, with primary care clinicians with a valid National Provider Identifier, a unique identifier assigned to each practicing clinician in the U.S., excluding 11.6% (n=4,769,762) of the beneficiaries. We defined primary care clinicians as physicians with a broad specialty designation of primary care, or physicians with a primary specialty of general gynecology (whom women may use as primary source of care), in addition to nurse practitioners and physician assistants.

Since the Medicare data do not designate a patient’s primary source of care directly, we used the established method of indirect attribution based on the plurality of office visits (14); in case of ties, patients were attributed to more than one clinician. Further, we excluded clinicians with less than 25 attributed patients to establish accurate estimates, and clinicians with more than 50% of patients carrying a diagnosis of cognitive impairment (MCI or dementia), as those were assumed to practice memory care, resulting in an analytic sample of 226,756 primary care clinicians.

To identify the primary care practices, we mapped primary care clinicians to practices via the tax identification number (TIN), under which the clinicians most frequently reported in Medicare Part B non-institutional claims. Prior research shows that 92.8% of individual clinicians reported under “a single TIN or under one dominant TIN” (15). Then, we attributed patients to practices using the same method described above, based on the plurality of office visits, which leads to an analytic sample of 54,597 practices.

Data were accessed through the Centers for Medicare & Medicaid Services Virtual Research Data Center. The study protocol and data protection procedures were approved by the Institutional Review Board at the University of Southern California (UP-21-00441), under expedited review and with a waiver for informed consent and Health Insurance Portability and Accountability Act authorization. Data were processed and analyzed using SAS 7.15 (SAS Institute Inc) and Stata 16 (StataCorp LLC).

### Identification of MCI Diagnosis in Claims Data

Beneficiaries diagnosed with MCI were identified based on the ICD-10-CM diagnosis code G31.84 (mild cognitive impairment of uncertain or unknown etiology). We required 2 claims with this code on separate days in the three-year window from 2017 to 2019, following the structure of the CCW algorithm for a dementia diagnosis (16). The diagnosis did not have to appear on a claim submitted by the primary care clinician to whom the patient was attributed, because it is possible that the diagnosis was made after referral to a specialist.

Claims for both MCI and dementia were found in 10.5% of individuals with either diagnosis, and we adjudicated those cases based on the following assignment rules: If a person was uniquely assigned to either MCI or dementia during the midpoint year, we used that assignment. If not, we based the assignment on the latest claim with a diagnosis of MCI or dementia in the midpoint year. If neither diagnosis was documented during the midpoint year, we based the assignment on the claim closest to the midpoint year or, for ties, in the earlier year. These rules assigned 39.6% of cases to MCI and 60.4% to dementia.

### Development and Validation of Model to Generate Expected Rates

We developed a predictive model, using HRS data from 2000 to 2016 (17), and applied it to the Medicare data to generate expected rates of MCI diagnosis.

As mentioned above, the HRS routinely administers cognitive assessments to its participants and collects information from proxy respondents when the primary participants cannot complete the survey. We applied the Langa-Kabeto-Weir algorithm (17, 18) developed by cross-walking cognitive scores to in-person clinical assessments using a subsample of HRS participants, to categorize all HRS participants aged 65 and older as: cognitively normal, having cognitive impairment but no dementia (CIND, representing MCI), or having dementia. The data years were limited to 2016 and earlier because the RAND-HRS longitudinal files do not include cognitive variables for newer HRS waves (19).

Because the same predictive model, predicting CIND (versus cognitively normal) using HRS data, must be applied to the Medicare data to allow generation of expected rates in the Medicare population, we used a probit model and chose the set of predictors that were available in both data sources with identical definitions. This includes sex, age groups (65–69, 70–74, 75–79, 80–84, and ≥85 years), race and ethnicity (non-Hispanic [NH] White, NH Black, Hispanic, and NH Other), dual eligibility status (individuals covered by both Medicare and Medicaid versus Medicare only), in addition to a linear trend for year to account for the secular decline in impairment incidence (20). The same modeling strategy was used to predict dementia (versus cognitively normal) as the computation of the expected rates of MCI must also factor in the probability of an individual having dementia. Details of this computation, including our exploration of the uncertainty in the expected rates using bootstrapping, which was found to be minor, are described in eMethod 1 in the Supplement.

While the calibration was conducted using the 2000 to 2014 HRS data, we evaluated the prediction accuracy of the derived regression weights (eTable 1 in the Supplement) using the 2016 data. Specifically, we calculated probabilities of an individual having CIND based on the predictive model (eMethod 1), and derived the receiver operating characteristic curve (eFigure 1) and prediction accuracy measures (eTable 2) by cross-walking such probabilities to the grouping identified based on cognitive assessment and proxy data. With sampling weights, we also compared the rates of having CIND based on predicted probabilities versus based on the cognitive and proxy data (eTable 2). With adequate prediction accuracy, the predictive model was consequently applied to the aforementioned Medicare sample to generate the expected diagnosis rates for MCI for each clinician’s or practice’s patient panel. Additional details of the predictive model can be found elsewhere (21)

### Calculation of Detection Rates with Inference

Clinician’s detection rates were calculated as the ratio between the observed rates based on diagnoses documented in the claims and encounter data and the expected rates described above, providing a measure for potential gaps in diagnoses. Such observed to expected ratios (O/E ratio) are frequently used in quality measurement (22), and can be interpreted as the proportion of expected cases that were diagnosed.

The interpretation of the detection rate requires a determination of whether or not the observed diagnosis rate of a clinician or practice is significantly different from the expected rate, or put differently, whether the detection rate is significantly different from 1. Adams and colleagues (23) have developed a statistical approach to make inference of an O/E ratio, when the observed and expected values are computed based on a continuous variable, in their case physician cost. Following their approach, we developed a method to make inference when the observed and expected values are computed based on a binary variable (e.g., receiving an MCI diagnosis or not), as detailed in eMethod 2.

Conceptually, the method involves calculating the 95% confidence interval (CI) around the observed diagnosis rate of each clinician or practice to account for sampling error. The inference of whether the detection rate is significantly different from 1 is then made by comparing the 95% CI for the observed rate to the expected rate. If the upper bound of the 95% CI around the observed rate is lower than the expected rate, it implies the detection rate is significantly lower than 1 and therefore the clinician or practice may be under-diagnosing compared to what’s expected; conversely, if the lower bound is higher than the expected rate, it suggests the detection is significantly more than what’s expected; and a 95% CI of the observed rate covering the expected rate indicates adequate detection.

## Results

Among the 226,756 included primary care clinicians, 25.5% practiced internal medicine, 35.5% specialized in family practice, and 36.1% served as nurse practitioners or physician assistants, leaving 0.4% practicing geriatric medicine and 2.6% practicing obstetrics/gynecology.

**Figure 1:**
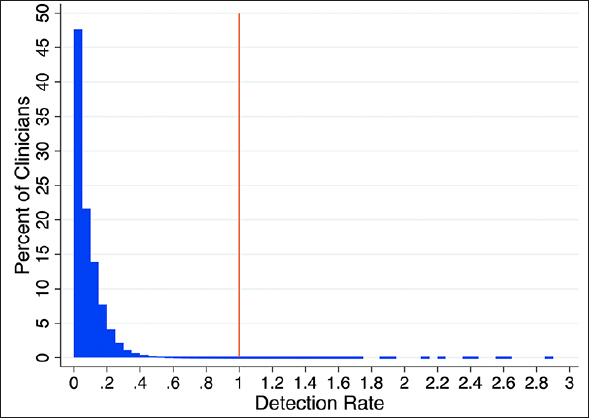
Distribution of the detection rates (the ratio of observed over expected rates) for clinicians on mild cognitive impairment, with reference to ratio of 1 as the ideal detection rate (red vertical line)

Table 1 shows the distributions of the observed and expected rates for the diagnosis of MCI, as well as the detection rates. Over a quarter of clinicians and practices did not have a single patient with diagnosed MCI in their panel with a median observed diagnosis rate of 0.01 (mean=0.01, SD=0.02, interquartile range [IQR]=0.00-0.02 for both clinicians and practices). The expected rates, however, suggested that around a fifth of their attributed patients were expected to be diagnosed on average (mean=0.19, SD=0.05, IQR=0.16-0.21 for clinicians; mean=0.20, SD=0.06, IQR=0.16-0.23 for practices). As a result, the average detection rates for clinician and practices were around 0.08 (SD=0.10, IQR=0.00-0.12 for clinicians; SD=0.10, IQR=0.00-0.10 for practices). Figure 1 shows a histogram of clinicians' detection rates for the diagnosis of MCI, with a heavy concentration close to 0.

**Table 1:** Distribution of the observed, expected, and detection rates for MCI diagnosis in primary care

					**Percentile**		
	**Mean**	**SD**	**5th**	**25th**	**50th/Median**	**75th**	**95th**
Clinician (N=226,756)							
Observed rate	0.01	0.02	0.00	0.00	0.01	0.02	0.05
Expected rate	0.19	0.05	0.14	0.16	0.18	0.21	0.30
Detection rate	0.08	0.10	0.00	0.00	0.06	0.12	0.25
Practice (N=54,597)							
Observed rate	0.01	0.02	0.00	0.00	0.01	0.02	0.05
Expected rate	0.20	0.06	0.14	0.16	0.19	0.23	0.32
Detection rate	0.08	0.10	0.00	0.00	0.05	0.10	0.23

Abbreviations: MCI, mild cognitive impairment. Notes: The detection rate is calculated as the ratio of observed over expected rates.

Based on inference for the detection rate, we classified the clinicians or practices into three groups: those whose observed rate was significantly lower than the expected rate, no different from the expected rate, or significantly higher than the expected rate, after accounting for the sampling errors. Table 2 shows that 99.9% of the clinicians and 99.8% of the practices had an observed rate significantly lower than the expected rate for diagnosis of MCI.

**Table 2:** Distribution of the diagnostic performance rating for MCI

**Observed rate is**…^a^	**Count**	**Percent**
Clinician (N=226,756)		
Lower than expected rate	226,518	99.9%
No different from expected rate	189	0.1%
Higher than expected rate	49	0.0%
Practice (N=54,597)		
Lower than expected rate	54,510	99.8%
No different from expected rate	62	0.1%
Higher than expected rate	25	0.0%

Abbreviations: MCI, mild cognitive impairment. a.The inference is made by comparing the 95% CI of the observed rate with the expected rate.

Broken down by clinician’s specialty (Table 3), those practicing geriatric medicine on average had an observed rate 2.5–3.7 folds of those practicing other specialties; their detection rate (mean=0.19, SD=0.16) was also more than double of the rates for other clinicians (mean ranges from 0.07–0.09). Despite their relatively better diagnostic performance for MCI, 99.7% of geriatric clinicians still had an observed rate significantly lower than the expected rate.

**Table 3:** Distribution of the observed, expected, and detection rates for MCI diagnosis by clinician’s specialty

**Specialty**	**Number of Clinicians**	**Observed Rate**		**Expected Rate**		**Detection Rate**	
		**Mean**	**SD**	**Mean**	**SD**	**Mean**	**SD**
Internal medicine	57729	0.02	0.02	0.21	0.05	0.09	0.09
Geriatric medicine	924	0.04	0.04	0.24	0.05	0.19	0.16
Family practice	80486	0.01	0.02	0.19	0.05	0.08	0.09
Obstetrics/gynecology	5785	0.01	0.02	0.16	0.04	0.07	0.11
Nurse practitioner/physician assistant	81832	0.01	0.02	0.18	0.04	0.07	0.11

## Discussion

The current study used Medicare administrative data for the full population aged 65 and older, including Medicare fee-for-service and Medicare Advantage Plans, to estimate detection rates for MCI in patients under the care of primary care clinicians. The results suggest that MCI remains vastly underdiagnosed with average detection rates of 0.08 for both clinicians and practices, implying that only about 8% of expected cases are diagnosed on average. Additionally, only one tenth of a percent of clinicians and practices have diagnosis rates within the expected range. This finding of substantial failure to diagnose MCI is in line with the above-mentioned findings by White et al (7) and Savva et al (8), who estimated detection rates of 11.4% and 15%, respectively, based on self-report.

This is particularly in contrast to recent studies (12) showing that detection rates for dementia have converged to the expected numbers, which suggests that clinicians have become more attentive to cognitive decline in general but not yet as much to its early stages. This finding is concerning not only because patients might not get identified for a disease-modifying AD treatment in time (6), but also because numerous causes of MCI - such as hypothyroidism and medication side effects - are reversible, and the condition itself can be stabilized by lifestyle modification interventions (24).

The reasons for low detection rates for MCI are manifold and include clinician-level factors, such as limited skill and limited confidence in ability to diagnose cognitive impairment (25), time constraints during routine office visits (26), and uncertainty about benefit of a diagnosis (26), compounded by system-level factors, such as lack of EHR integration and dedicated reimbursement for brief cognitive tests (27). A misinterpretation of the U.S. Preventive Services Taskforce statement (28) that the evidence is insufficient to recommend for or against routine cognitive screening as ruling against such screening might also contribute to reluctance to follow up on cognitive complaints.

Several expert groups (26, 29, 30) have made recommendations for improving the detection of MCI in primary care. These recommendations include (a) better education and training (29), tools such as simple cognitive assessment tests, order sets and practice guidelines, (b) consistent and reliable reimbursement that is commensurate with the effort and time required to evaluate cognitive state (26), and (c) accountability schemes based on detection rates, such as those currently used in the U.K. (30, 31, 32). A recent full approval (3) of lecanemab on July 6, 2023 adds to the urgency with which such recommendations need to be implemented.

### Limitations

Our findings should be interpreted within the context of the study limitations. First, we acknowledge that a predictive model based on demographic information alone has only moderate accuracy. Second, we estimated expected prevalence of MCI based on cognitive test scores, which is not the same as a true clinical diagnosis. However, our predicted number of 8.06 million cases in the U.S. Medicare population is close to the 7.95 million predicted based on a widely recognized meta-analysis by Petersen et al. (33) Third, our algorithm to ascertain MCI in administrative data is modeled after the validated CCW algorithm for dementia, but should undergo its own validation, including the selection of diagnosis codes. Fourth, our assignment rules for cases with claims for both MCI and dementia may have misattributed cases. However, even attributing all of those cases to MCI would have increased the average detection rate by only 0.5 percentage points. Lastly, diagnoses may have been communicated but not documented in claims data because of concerns about stigma and loss of driver’s license, but lack of documentation would still represent a problem, as it might limit the clinician’s ability to prescribe medications and connect patients with support services.

## Conclusion

Overall, our findings represent—to our knowledge—the first assessment of the rate at which U.S. primary care clinicians detect MCI in the full Medicare population. These results point to a need to take measures that improve early detection of cognitive impairment, particularly in light of the emergence of disease-modifying treatments for Alzheimer’s disease as the most common etiology for MCI.

### Electronic supplementary material

Appendix

## Abbreviations

*AD*: *Alzheimer’s Disease*
*MCI*: *mild cognitive impairment*
*HRS*: *Health and Retirement Study*
*CCW*: *Chronic Conditions Data Warehouse*
*TIN*: *tax identification number*
*CIND*: *cognitive impairment but no dementia*
*NH*: *non-Hispanic*
*O/E ratio*: *observed to expected ratios*
*CI*: *confidence interval*
*IQR*: *interquartile range*

## Notes

*Ethical standards:* Ethical approval was given by the local ethnical committee at the University of Southern California, and the research was conducted in accordance with the Helsinki Declaration.
