# Supplementary material for: Detection Rates of Mild Cognitive Impairment in Primary Care for the United States Medicare Population
Source: J Prev Alzheimers Dis. 2023 Oct 24;11(1):7–12. doi: 10.14283/jpad.2023.131 (PMC10995024; doi:10.14283/jpad.2023.131)
Supplement: Supplementary file 1 — Appendix [file 42414_2023_284_MOESM1_ESM.docx]

# Appendix

# **eMethod 1.** Derivation of expected probabilities of having MCI, dementia, or normal cognitive state from probit model predictions

**eMethod 2.** Inference of O/E ratio when the observed and expected values are computed based on a binary outcome.

# **eTable 1.** Probit model estimates using 2000 to 2014 HRS data from respondents aged 65 and older

# **eTable 2.** Validation of predicted rates against observed rates using 2016 HRS data among respondents aged 65 and older (N=9,808)

# **eFigure 1.** Area under the receiver operating characteristic curve when the predicted probabilities of having MCI or dementia (vs being cognitively normal) are compared to the cognitive states determined by cognitive assessments and information reports, using 2016 HRS data from respondents aged 65 or older

## **eMethod 1. Derivation of expected probabilities of having MCI, dementia, or normal cognitive state from probit model predictions**

Because the three cognitive states are all inter-dependent, we used probit models to separately predict having mild cognitive impairment (MCI) vs being cognitively normal (CN) and having dementia vs CN. In other words, the predicted values from each equation, with previously calibrated regression weights, reflect the expected proportion of MCI among MCI and CN subsamples and expected proportion of dementia among dementia and CN subsamples. To obtain the expected rates of the 3 cognitive states over the entire sample, additional conversion is needed, and we show the mathematical derivation below.

Let $p^{cn}$, $p^{mci}$, and $p^{dem}$ represent the expected proportions of CN, MCI, and dementia, respectively, over the entire sample, and $e^{mci}$ and $e^{dem}$ denote the predicted probabilities from the probit models. We then have

$e^{mci}=$ $p^{mci}/(p^{mci}+p^{cn})$ --- (1)

$e^{dem}=$ $p^{dem}/(p^{dem}+p^{cn})$. --- (2)

With simple transformation, these can be rewritten as:

$p^{mci}=p^{cn}\cdot e^{mci}/\left( 1-e^{mci} \right)$ --- (3)

$p^{dem}=p^{cn}\cdot e^{dem}/\left( 1-e^{dem} \right)$. --- (4)

Given that

$p^{cn}+p^{mci}+p^{dem}=1$, --- (5)

plugging (3) and (4) into (5), and solving the equation will result in:

$p^{cn}=\frac{\left( 1-e^{mci} \right)\left( 1-e^{dem} \right)}{e^{dem}\left( 1-e^{mci} \right)+e^{mci}\left( 1-e^{dem} \right)+\left( 1-e^{mci} \right)\left( 1-e^{dem} \right)}$ --- (6)

Further plugging the computed $p^{cn}$ into (3) and (4) will obtain $p^{mci}$ and $p^{dem}$, respectively.

*Noise due to inaccurate prediction.* Because $e^{mci}$ and $e^{dem}$ are predicted values based on regression estimates, one concern is that the inaccuracy in prediction may induce noise to the resulted expected proportions described above. Thus, we evaluate the magnitude of such inaccuracy by perturbing the estimates of the predicted model (eTable 1) around their 95% confidence intervals. In other words, we account for the fact that the true value of a parameter may not be identical to the point estimate, but rather, somewhere in the 95% confidence interval.

Specifically, we leverage the bootstrapping approach, a well-established approach to evaluate the noise due to imperfect estimation, and randomly draw parameter values from the estimated confidence intervals. We then apply these values to compute $e^{mci}$ and $e^{dem}$ of each demographic characteristic group $k$, e.g., the predicted probability of a Hispanic female aged 60-64 who had Medicare only having MCI (vs. not) or dementia (vs. not). By repeating this process for 1000 times, we compute the bootstrap standard error of group $k$’s predicted probability, accounting for the imperfect regression estimates.

For each characteristic group, the resulted bootstrap standard error is no more than 10% of the predicted probability computed using the point estimates, i.e., $e^{mci}$ and $e^{dem}$ we used in our analysis. The relatively larger standard errors are seen on characteristic groups with a smaller population share, such as racial/ethnic minorities aged 85 and older who are dually eligible for Medicare and Medicaid.

In summary, these results suggest that the noise due to inaccurate regression estimation has limited impact on our computation and inference of O/E ratio.

**eMethod 2. Inference of O/E ratio when the observed and expected values are computed based on a binary outcome.**

Here, we derive an inference approach testing whether our detection rate measure, an observed-over-expected (O/E) ratio, is significantly different from 1 or not when the observed and expected values are based on a binary outcome. This approach is generally applicable with or without attribution of patients to a specific unit (e.g., clinician or practice). Following the current study’s context, we describe our approach considering the outcome of diagnosis with MCI or not among patients who are attributed to a clinician or practice.

Under the assumptions that each patient’s diagnosis status is independent of another patient’s status and patients attributed to a specific unit reflect a random sample of the entire patient population, whether a patient receives a diagnosis follows a Bernoulli process, in which the chance of getting a diagnosis is fixed among patients with similar characteristics. In this study, we chose the same list of patient characteristics as those used in estimation of the expected rate (sex, age, race and ethnicity, and dual eligibility status), and estimated the population-level diagnosis rate for patients with the same characteristics (e.g., Hispanic females aged 60-64 who had Medicare only).

Let $p_{k}$ denote the population-level diagnosis rate for patients with characteristic group $k$, when $k=1,\ldots,K$. For a specific unit $i$ (clinician or practice), its attributed patient pool includes $n_{ik}$ many patients in characteristic group $k$, among whom $y_{ik}$ received a diagnosis. It can be derived easily that $y_{ik}\sim Binomial(n_{ik},p_{k})$, each representing one characteristic group among the attributed patients. Consequently, the total number of attributed patients who received a diagnosis can be characterized using a mixture of such Binomial distributions.

Specifically, let $N_{i}=\sum_{k} n_{ik}$ represent the total number of patients attributed to the unit $i$. The unit’s observed diagnosis rate is essentially $O_{i}=\sum_{k} y_{ik}/N_{i}$, and its variance can be written as $var\left( O_{i} \right)=\frac{1}{N_{i}^{2}}\sum_{k} n_{ik}p_{k}\left( 1-p_{k} \right)$. Taking the square root of the variance gives the standard error of $O_{i}$, which can then be used to derive the test statistic or confidence interval for inference.

Since the sampling error is the primary concern of noise when making inference about the O/E ratio, we further assume that the noise in the observed rate is much larger than the noise in the expected rate. Testing a null hypothesis of O/E=1 can then be framed as testing whether the observed rate, with the aforementioned standard error, is significantly different from the expected rate.

## **eTable 1. Probit model estimates using 2000 to 2014 HRS data from respondents aged 65 and older**

|  | **MCI^a^** | | **Dementia^a^** | |
| --- | --- | --- | --- | --- |
|  | *b* | 95% CI | *b* | 95% CI |
| Constant | -1.3078 | (-1.343--1.272) | -2.0242 | (-2.083--1.966) |
| Sex: male^b^ | 0.1171 | (0.084-0.150) | 0.0293 | (-0.019-0.077) |
| Age groups^c^ |  |  |  |  |
| 70-74 | 0.1969 | (0.162-0.232) | 0.2835 | (0.227-0.341) |
| 75-79 | 0.4460 | (0.408-0.484) | 0.6636 | (0.602-0.725) |
| 80-84 | 0.7022 | (0.659-0.746) | 1.0693 | (1.004-1.135) |
| ≥85 | 1.0780 | (1.029-1.127) | 1.7013 | (1.633-1.770) |
| Race & ethnicity^d^ |  |  |  |  |
| Non-Hispanic Black | 0.7421 | (0.693-0.791) | 0.8225 | (0.756-0.889) |
| Hispanic | 0.6229 | (0.560-0.686) | 0.5554 | (0.466-0.645) |
| Other | 0.2565 | (0.135-0.378) | 0.3116 | (0.149-0.474) |
| Dually eligible^e^ | 0.5608 | (0.506-0.616) | 1.0198 | (0.954-1.085) |
| Year^f^ | -0.0117 | (-0.015--0.009) | -0.0182 | (-0.022--0.014) |
| Pseudo R-squared | 0.0962 |  | 0.2447 |  |
| N^g^ | 77,206 |  | 68,612 |  |

^a^Compared to being cognitively normal. The standard errors are clustered at the household level.

^b^Compared to female.

^c^Compared to respondents aged 65 to 69 years.

^d^Compared to non-Hispanic White.

^e^Compared to Medicare-only beneficiaries.

^f^Year recoded from 2000 to 0, from 2002 to 2, etc, respectively.

^f^Sample size reflects cumulative numbers of observations across 2000 to 2014.

Abbreviations: HRS, Health and Retirement Study; MCI, mild cognitive impairment.

## **eTable 2. Validation of predicted rates against observed rates using 2016 HRS data among respondents aged 65 and older (N=9,808)**

|  | **MCI** | | **Dementia** | |
| --- | --- | --- | --- | --- |
|  | Rate | 95% CI | Rate | 95% CI |
| Observed^a^ | 0.1753 | (0.167-0.184) | 0.0781 | (0.072-0.084) |
| Predicted^b^ | 0.1764 | (0.174-0.179) | 0.0816 | (0.079-0.084) |
| AUC^c^ | 0.7149 |  | 0.8354 |  |
| Sensitivity^d^ | 47.09% |  | 57.35% |  |
| Specificity^d^ | 81.43% |  | 89.10% |  |
| PPV^d^ | 43.43% |  | 45.37% |  |
| NPV^d^ | 83.56% |  | 92.98% |  |
| Accuracy^d^ | 73.45% |  | 84.77% |  |

^a^Computed based on the weighted proportion of 2016 HRS respondents who were classified to having MCI or dementia based on their cognitive assessments or informant reports.

^b^Computed based on the weighted average of the respondents’ predicted probabilities of having MCI or dementia by applying the estimates from the probit calibration (using 2000-2014 data) to respondents in 2016 and converting the predicted values as shown in Appendix A.

^c^Area under the receiver operating curve when the predicted probabilities of having MCI or dementia (vs being cognitively normal) are compared against the cognitive states determined by cognitive assessments or informant reports.

^d^Sensitivity, specificity, PPV, NPV, and accuracy is based on a cutoff of 0.3.

Abbreviations: AUC, area under the curve; MCI, mild cognitive impairment; NPV, negative predictive value; PPV, positive predictive value.

## **eFigure 1. Area under the receiver operating curve when the predicted probabilities of having MCI or dementia (vs being cognitively normal) are compared to the cognitive states determined by cognitive assessments and information reports, using 2016 HRS data from respondents aged 65 or older**

## MCI vs being cognitively normal

## Dementia vs being cognitively normal

Abbreviations: HRS, Health and Retirement Study; MCI, mild cognitive impairment; ROC, receiver operating characteristic.
